# Clinicopathologic characteristics and prognostic factors of 63 gastric cancer patients with metachronous ovarian metastasis

**DOI:** 10.7497/j.issn.2095-3941.2013.02.004

**Published:** 2013-06

**Authors:** Qiang Feng, Wei Pei, Zhao-Xu Zheng, Jian-Jun Bi, Xing-Hua Yuan

**Affiliations:** Department of Abdominal Surgery, Cancer Institute and Hospital, Chinese Academy of Medical Science and Peking Union Medical College, Beijing 100021, China

**Keywords:** Gastric neoplasms, ovary, metastasis, prognosis

## Abstract

**Objective:**

This study aims to explore the clinicopathologic characteristics and prognostic factors of gastric cancer patients with metachronous ovarian metastasis.

**Methods:**

Clinicopathologic data were collected from 63 post-operative gastric cancer patients with metachronous ovarian metastasis. The patients were admitted to the Cancer Institute and Hospital, Chinese Academy of Medical Science and Peking Union Medical College between January 1999 and December 2011. A log-rank test was conducted for survival analysis. Possible prognostic factors that affect survival were examined by univariate analysis. A Cox regression model was used for multivariate analysis.

**Results:**

The incidence of ovarian metastasis was 3.4% with a mean age of 45 years. Up to 65.1% of the patients were pre-menopausal. The mean interval between ovarian metastasis and primary cancer was 16 months. Lowly differentiated carcinoma ranked first in the primary gastric cancers. The majority of lesions occurred in the serous membrane (87.3%). The metastatic sites included N_2-3_ lymph nodes (68.3%), bilateral ovaries (85.7%), and peritoneal membrane (73%). Total resection of metastatic sites was performed (31.7%). The overall median survival was 13.6 months, whereas the overall 1-, 2-, and 3-year survival rates were 52.5%, 22.0%, and 9.8%, respectively. The 5-year survival rate was zero. Univariate analysis showed that the patient prognosis was correlated with metastatic peritoneal seeding, vascular tumor embolus, range of lesion excision, and mode of comprehensive treatment with adjuvant chemotherapy (*P*<0.05). Multivariate analysis indicated that metastatic peritoneal seeding was an independent prognostic factor for gastric cancer patients with ovarian metastasis (*P*<0.01).

**Conclusion:**

Effective control of peritoneal seeding—induced metastasis is important for improving the prognosis of gastric cancer patients with ovarian metastasis.

## Introduction

Gastric cancer is one of the most common malignant neoplasms. Although the diagnosis and treatment have improved significantly in recent years, about 50% of the patients recrudesce and develop distant metastasis after treatment. Ovaries are the common metastasis site for many malignant neoplasms. The incidence rate of postoperative ovarian metastasis of female gastric cancer patients ranges from 2.7% to 6.7%^1^^,^^2^. However, autopsy results show that the incidence rate of ovarian metastasis ranges from 33% to 41%^3^^,^^4^. Currently, effective treatment methods for gastric cancer ovarian metastasis are lacking. In addition, research on metachronous ovarian metastasis after gastric cancer treatment is insufficient. This study aims to discuss further the clinicopathologic characteristics, treatment methods, and prognosis through retrospective analysis of the clinical data of gastric cancer patients with ovarian metastasis treated in our hospital.

## Patients and methods

### Clinical data

From January 1999 to December 2011, 1,856 female gastric cancer patients were admitted and treated at the Chinese Academy of Medical Science Tumor Hospital. Among these patients, 63 patients 21 to 70 years old (mean age: 45 years) were histopathologically proven to have metachronous ovarian metastasis by secondary surgery. Among these 63 patients, 9 were primary gastric cancer patients that had excision in other hospitals with the consultation result borrowed from other hospitals’ tissue sample as histopathologic files, and 54 had their histopathologic diagnosis of surgical samples conducted in our hospital. These patients were grouped according to primary gastric cancer lesion: gastric sinus (40 cases), gastric body (18 cases), and cardia and fundus (5 cases). They were also classified according to their ovarian metastasis neoplasms: unilateral (9 cases) and bilateral (54 cases). The common clinical manifestations were hypogastric pain, abdominal mass, irregular vaginal bleeding, and so on.

### Diagnostic methods

The main diagnostic methods include gastroscopy, B ultrasound, computed tomography (CT), nuclear magnetic resonance (NMR), and so on. The tumor marker examination for neoplasms includes CEA, CA199, CA724, and CA125. All patients of this group received treatment for gastric cancer and ovarian metastasis. For primary cancer, 43 cases underwent distal subtotal gastrectomy, 4 underwent proximal subtotal gastrectomy, and 16 underwent total gastrectomy. Ovarian metastatic neoplasms: 20 cases underwent total lesion excision as per the extent of disease and 43 cases underwent palliative excision because of the wide metastasis in peritoneal membrane. Among the cases, 9 cases underwent unilateral excision, 54 cases underwent bilateral excision, 21 cases underwent uterus combination excision, and 3 cases underwent limited peritoneal membrane metastasis combination excision. Auxiliary treatment includes chemotherapy after ovarian metastasis cancer surgery. The chemotherapy medicine mainly includes fluorouracil, oxaliplatin/cisplatin, calcium folinate, docetaxel, and so on.

### Follow-up

All the cases in this group underwent follow-up visits mainly via outpatient review, telephone, follow-up mail, and so on. The recorded survival time is from diagnosed ovarian metastasis until death or until final follow-up.

### Statistical analysis

All data were statistically analyzed with SPSS 17.0 software statistical package. The survival rate was calculated with the Kaplan-Meier method, and the Log-rank test was conducted to analyze survival rate difference among groups. Cox regression model was applied for multi-factor prognosis analysis. Differences with *P*<0.05 were considered statistically significant.

## Results

### Clinical and histopathologic characteristics

In this study, 1,856 female gastric cancer patients underwent excision, among which 63 (3.4%) developed metachronous ovarian metastasis. The interval to metastasis was 2 months to 20 years after gastric cancer surgery, with an average of 16 months. Up to 46 patients were in their 50s (73.0%), and 41 patients (65.1%) were pre-menopausal. Ovarian metastasis patients had corresponding symptoms after primary cancer surgery or definitive diagnosis after regular review with imaging tests. In this group, imaging results showed that 18 cases with metastatic ovarian lesions were cystic or cystic and solid disease, among which 6 (9.5%) were diagnosed as physiologic enlargement by mistake during B ultrasound/CT regular review in the early period after gastric cancer surgery. Up to 39 patients had corresponding serum tumor marker check, among which 7 (18%) were CEA positive, 15 (38.5%) were CA199 positive, 9 (23.1%) were CA724 positive, and 24 (61.5%) were CA125 positive. Any one of the previous three markers was increased at the same time with CA125 in 20 cases (51.3%). A total of 11 cases (28.2%) were negative for all markers.

The histopathologic result showed that primary gastric cancer was mainly poorly differentiated. Among the 63 patients, 53 (84.1%) had primary gastric carcinoma, 55 (87.3%) had invaded serosa, and 43 (63.8%) had N_2-3_ lymph node metastasis. The patients showed different histopathologic types of ovarian metastasis: gastric signet ring cell carcinoma (38 cases), tubular adenocarcinoma (21 cases), and mucinous adenocarcinoma (4 cases). Unilateral metastasis was found in 9 cases, and bilateral metastasis was found in 54 cases. The diameter of neoplasms ranged from 2 to 20 cm, with an average diameter of 8.4 cm. Up to 19 patients had neoplasm diameter >10 cm. Vascular tumors were found in 28 cases, and metastatic peritoneal seeding was found in 46 cases.

### Follow-up

The follow-up period was from January 1999 to October 2012. The overall median survival period was 13.6 months, with 49 mortalities. The 1-, 2-, and 3-year survival rates were 52.5%, 22.0%, and 9.8%, respectively. The 5-year survival rate was zero.

### Influence on prognostic factors

**Figure 1:**
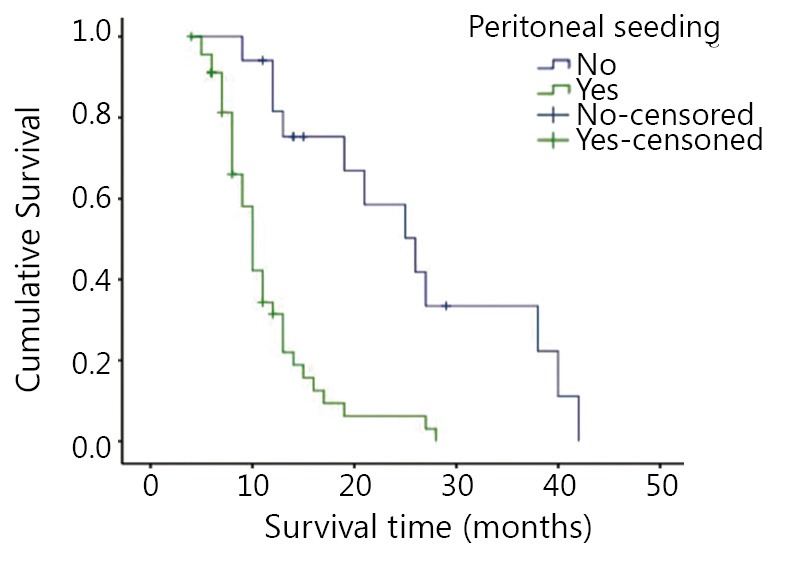
Survival curves for patients with/without metastatic peritoneal seeding.

The single-factor survival analysis showed that peritoneal seeding, metastatic lesion, vascular tumor emboli, lesion excision range, and the treatment mode of auxiliary chemotherapy influenced patient prognosis (*P*<0.05). Age, menstrual history, gastric cancer lymph node metastasis, the interval between metastasis lesion and primary lesion, size of metastasis, metastasis site, and metastatic histopathologic type had no significant relationship with prognosis (**Table 1**). The Cox regression model multi-factor analysis result showed that metastatic peritoneal seeding was the determining factor that influenced the prognosis of gastric cancer patients with ovarian metastasis. Compared with gastric cancer ovarian metastasis patients with metastatic peritoneal seeding, those without metastatic peritoneal seeding had significant extended survival period (*P*<0.01, **Table 2** and **Figure 1**).

**Table 1:** Univariate analysis of prognostic factors for gastric cancer patients with ovarian metastasis

Characteristics	*n* (%)	1-year survival rate (%)	3-year survival rate (%)	Median survival period (months)	χ^2^	*P*
Age, years					0.004	0.952
≤50	46 (73.0)	55.8	30.6	13.1		
>50	17 (27.0)	44.7	19.1	10.3		
Menstrual history					0.070	0.792
Pre-menopausal	41 (65.1)	57.1	5.9	12.6		
Post-menopausal	22 (34.9)	42.3	18.1	10.4		
Lymph node metastasis of gastric carcinoma					3.013	0.083
N_0-1_	20 (31.7)	72.6	16.8	12.9		
N_2-3_	43 (68.3)	43.4	5.1	11.2		
Interval between metastatic and primary foci (months)					0.159	0.690
≤6	21 (33.3)	42.8	15.3	9.8		
>6	42 (66.7)	56.8	8.5	13.3		
Ovarian metastasis					1.107	0.293
Unilateral	9 (14.3)	44.4	11.1	10.6		
Bilateral	54 (85.7)	54.3	11.5	11.5		
Size of metastatic foci (cm)					2.785	0.095
>10	44 (69.8)	56.9	11.5	12.8		
≤10	19 (30.2)	36.5	0	9.6		
Peritoneal seeding					19.719	<0.001
Yes	46 (73.0)	34.7	0	8.5		
No	17 (27.0)	88.2	30.5	20.7		
Histopathologic type of metastatic foci					1.010	0.604
Signet ring cell carcinoma	38 (60.3)	50.0	19.9	10.5		
Tubular adenocarcinoma	21 (33.3)	57.9	0	13.2		
Mucinous adenocarcinoma	4 (6.4)	50.0	0	10.2		
Vascular tumor thrombus of metastatic foci					2.676	0.049
Yes	28 (44.4)	44.8	6.2	9.6		
No	35 (55.6)	65.2	16.3	13.1		
Foci resection					23.327	<0.001
Total resection	20 (31.7)	89.5	27.3	19.4		
Palliative resection	43 (68.3)	33.7	0	8.8		
Therapeutic methods					5.772	0.016
Surgery alone	22 (34.9)	40.0	0	9.8		
Surgery plus chemotherapy	41 (65.1)	59.8	15.5	15.2		

**Table 2:** Multi-factorial analysis of prognostic factors for gastric cancer patients with ovarian metastasis

Clinical factors	β	SE	Wald	df	Sig.	Exp (B)	95% CI lower	95% CI upper
Peritoneal seeding	-2.393	0.888	7.259	1	0.007	0.091	0.016	0.521
Foci resection	1.167	0.672	3.020	1	0.082	3.213	0.861	11.981
Therapeutic mode	0.096	0.351	0.075	1	0.784	1.101	0.553	2.192
Vascular tumor thrombus of metastatic foci	-0.494	0.325	2.311	1	0.128	0.610	0.322	1.154

## Discussion

The ovarian metastatic tumor from gastric cancer is also called Krukenberg tumor, which was first reported by the German pathologist Friedrich Ernst Krukenberg in 1896. The reports on the incidence of gastric cancer ovarian metastasis are quite different. For 12 years, the incidence rate of metachronous ovarian metastasis was 3.4% in this research. Given that the research excluded from synchronous metastasis and non-surgical cases, the actual incidence of ovarian metastasis should be higher than the statistical result.

The pathogenesis of ovarian metastasis is unclear. Generally, there are three possible mechanisms: lymph node metastasis, hematogenous metastasis, and seeding metastasis. According to research, the ovarian reticular lymphatic tissue is rich and the cancer cells can pass to the waist lymph node through the retroperitoneal lymph node and paradoxic metastasis to the ovary, which is considered the most likely transfer method^5^. Kim *et al.*^2^ performed a multi-factor analysis on 690 female gastric cancer patients and showed that the incidence of ovarian metastasis is closely related to the extent of gastric cancer lymph node metastasis. Patients with metastases to more than six lymph nodes are more likely to have ovarian metastasis. In this group, the number of patients with N_2-3_ primary tumor lymph node metastasis (68.3%) was significantly higher than those without lymph node metastasis. Yamanishi *et al.*^6^ applied immunohistochemical staining and found that 57.0% of patients with ovarian metastasis have lymphatic involvement, which proves that ovarian metastases occur through lymph. Recently, hematogenous metastasis is receiving increasing support. Active premenopausal ovaries provide a more suitable growing environment for metastatic tumors because of their high hormone levels and rich blood supply. Therefore, young premenopausal patients are more likely to have ovarian metastasis^7^. When gastric cancer invades serous membranes, the cancer cells enter into the abdominal cavity and into the ovaries through the split holes formed during ovulation, becoming seeding metastasis. Seeding metastasis often spreads widely on the peritoneum and leads to unfavorable prognosis^8^. Most of the patients developed ovarian metastasis via this mechanism. Combination peritoneal seeding spread was observed in 73.0% of the patients. This finding was also the main reason for the unfavorable prognosis of this group. In fact, multiple metastases might coexist because the primary gastric cancers are mostly in the progressive stage.

Research has shown that metastatic tumors are often large, about 9 cm in diameter on average, whereas primary ovarian tumors are usually small^9^. The average tumor diameter among the patients was 8.4 cm, with 30.2% (19/63) larger than 10 cm. This result demonstrates that early ovarian metastasis is complicated. Koyama *et al.*^10^ reported that B ultrasound, CT, MR, and other examinations could reveal asymptomatic metastatic lesions during the early stage. For solid, lobulated, and contrast-enhanced ovarian lesions, the possibility for metastasis should always be considered. However, 18 (28.6%) of the 63 patients of this group had cystic or solid and cystic disease, as determined via B ultrasound and CT examination. Six cases were erroneously diagnosed as physiologic enlargement of the ovaries during the early stage of metastasis; these patients did not receive treatment in time. Therefore, we should pay attention to abnormal ovarian enlargement regardless of whether they are cystic or solid. Yada-Hashimoto *et al.*^4^ believed that a rapid increase in CA125 could be considered an auxiliary marker of ovarian tumors and Krukenberg tumors in the early stage. In this group, most patients were only tested for gastrointestinal tumor markers (CEA, CA199, or CA724) after gastric cancer surgery. Up to 39 cases were tested for CA125 when the ovarian lesion was found, and the results showed that CA125 increased to 61.5%, higher than the positive rate of any one marker. The CA125 test was indicative of Krukenberg tumor. Therefore, the surgeons should routinely test CA125 during the postoperative review of female gastric cancer patients.

The ovarian metastasis of gastric cancer is in the IV period, with unfavorable prognosis. Chemotherapy was previously recommended over surgical treatment. One of the main reasons for the unfavorable prognosis is the wide seeding metastasis of abdominal and pelvic peritoneum^11^^,^^12^. In recent years, with the improvement of diagnostic technology and the perfection of postoperative follow-up examination systems, some patients have been diagnosed during the early stage of ovarian metastasis, thereby extending the survival period through tumor excision. Jun *et al.*^13^ showed that the average survival period of gastric cancer patients with ovarian metastasis after secondary surgery is 18.8 months, among which the survival period of patients who underwent total excision of the metastatic lesion can reach 23.7 months. Early diagnosis and complete metastatic lesion excision can improve the survival rate of these patients. Cheong *et al.*^14^ analyzed clinical data from 34 ovarian metastasis cases. The result showed that patients who underwent total excision of the metastatic lesion had better prognosis than patients with residual tumors. Single-factor analysis of this group showed that patients who underwent total lesion excision had significantly longer median survival time compared with patients who underwent palliative lesion excision. This result was related with prognosis. However, multi-factor analysis showed that the lesion excision range did not influence the independent risk factors of prognosis.

The survival period of the patients in this group was 13.6 months. The 1-, 2-, and 3-year survival rates were 52.5%, 22.0%, and 9.8%, respectively, and the prognosis was unfavorable. The main reason was that most patients also developed metastatic peritoneal seeding, and only 32% of the patients underwent complete lesion excision. Multi-factor analysis showed that metastatic peritoneal seeding is the only independent risk factor that influenced patient prognosis in this group. Most studies^7^^,^^12^ showed that the tumor metastatic range and whether the tumor was resectable via radical excision were the main factors that influence prognosis. This report indicates that if the patients developed widespread peritoneal seeding metastasis simultaneously, surgery would not improve the prognosis. Jacquet *et al.*^15^ found that the accuracy of CT for the preoperative diagnosis of metastatic peritoneal seeding was only 50%. In recent years, laparoscopic exploration has been widely used in evaluating tumor resectability during the progressive stage. Performing ancillary imaging tests will more accurately identify ovarian metastasis patients^12^^,^^16^ who are suitable for surgical excision. At present, effective treatment methods for metastatic peritoneal spread are lacking. More reports about cytoreductive surgery for colorectal cancer patients have proven that it can effectively extend the survival time. However, reports about cytoreductive surgery for gastric cancer patients are insufficient. Bozzetti *et al.*^17^ showed that the median survival period was only  8 months to 11 months, with a surgical death rate of 2% to 7.1%. Recent studies^18^ have shown that hyperthermic intraperitoneal chemotherapy can treat metastatic peritoneal seeding and that its combination with cytoreductive surgery improves the survival rate of some peritoneal metastasis patients.

In summary, premenopausal female patients with gastric cancer should be vigilant about recurrent ovarian metastasis after surgery. Besides imaging tests, CA125 should be considered a routine marker after surgery for early diagnosis and subsequent treatment. In addition, prevention and effective treatment of widespread metastatic peritoneal seeding are the key points for improving patient survival.
